# Supplementary material for: COP1 Deficiency in BRAFV600E Melanomas Confers Resistance to Inhibitors of the MAPK Pathway
Source: Cells. 2025 Jun 25;14(13):975. doi: 10.3390/cells14130975 (PMC12249101; doi:10.3390/cells14130975)
Supplement: Supplementary file 1 [file cells-14-00975-s001.zip › cells-3663429-supplementary.pdf]

cells

MDPI

Supplementary Material

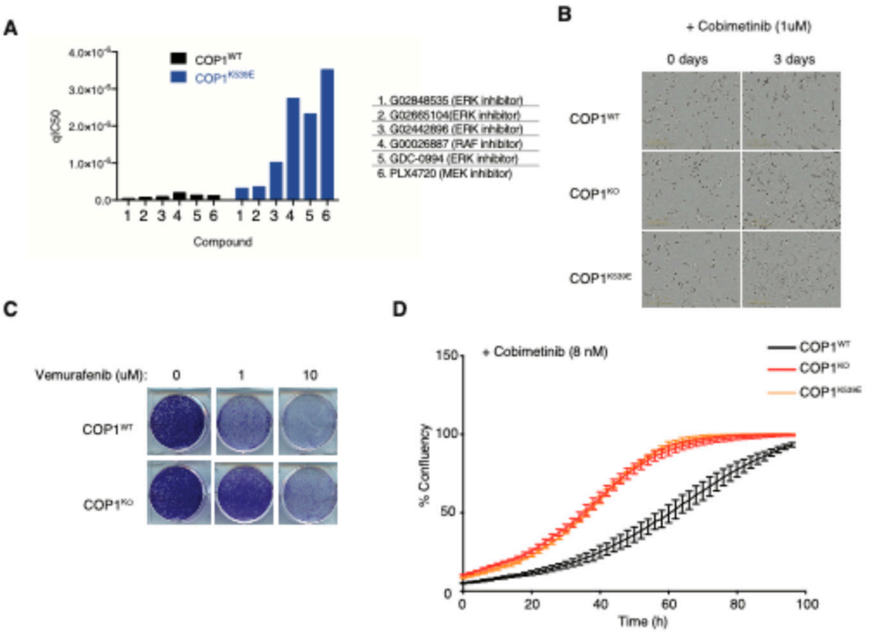

Figure S1. Deletion or mutational inactivation of COP1 mediates resistance to MAPK pathway inhibitors. (A) qIC50 values for MAPK pathway inhibitors in *COP1*^{WT} and *COP1*^{K539E} A375 cells. qIC50 is a ratio of IC50 values, which refers to the concentration of a drug or compound needed to cell viability by 50%. (B) Micrographs of *COP1*^{WT}, *COP1*^{KO} and *COP1*^{K539E} A375 cells at 0 and 3 days of treatment with 1 μM cobimetinib. (C) Crystal violet staining of *COP1*^{WT} and *COP1*^{KO} A375 cells at 3 days post treatment with 0, 1 or 10 μM vemurafenib. Results representative of 3 independent experiments. (D) Graphs indicate A375 cell proliferation upon treatment with 8 nM cobimetinib. Results representative of 3 independent experiments.

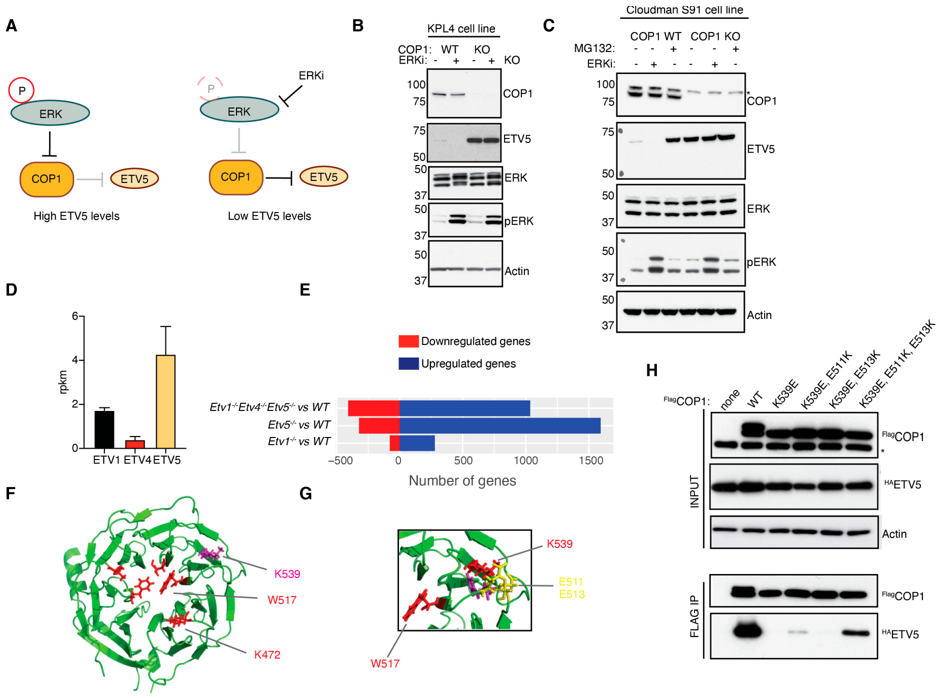

https://doi.org/10.3390/cells14130975

Figure S2. COP1^{K539E} fails to interact with and promote the degradation of ETV5. (A) Model for negative regulation of COP1 by ERK, leading to stabilization of ETV5. (B and C) Western blots of KPL4 cells or Cloudman S91 cells. Where indicated, cells were treated with 1 µM Vertex11e and/or 1 µM MG132 for 1 h. (D) *Etv1*, *Etv4* and *Etv5* mRNA expression in primary mouse melanocytes. RPKM, Reads Per Kilobase of transcript, per Million mapped reads. (E) Bars indicate the number of differentially expressed genes when comparing primary mouse melanocytes of the genotypes indicated. (F) Crystallography model of the COP1 WD40 domain, highlighting residues involved in substrate binding. (G) Magnified view of the select residues in (F). (H) Western blots of transfected 293T cells. IP, immunoprecipitation.

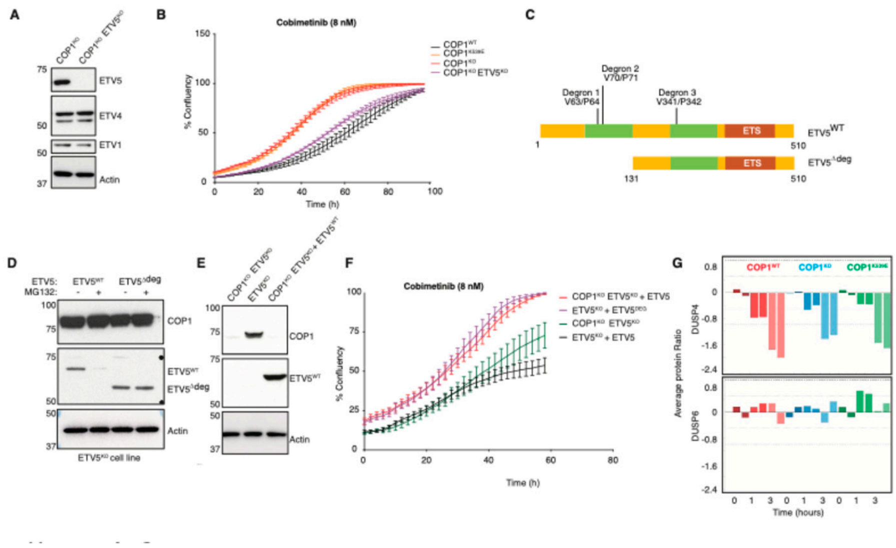

Figure S3. High levels of ETV5 correlate with resistance to MAPK pathway inhibitors. (A) Western blots of A375 cells. (B and F) Graphs indicate A375 cell proliferation upon treatment with 8 nM cobimetinib. Data are the mean ± 3 (n = 3 per condition). Results representative of 3 independent experiments. (C) Domain organization of human ETV5^{WT} and truncated ETV5$^{\Delta deg}$. (D) Western blots of A375 cells overexpressing either ETV5^{WT} or ETV5$^{\Delta deg}$, and treated with 1 µM MG132 for 1 h. (E) Western blots of A375 cells. (G) Bars indicate the relative abundance of DUSP4 or DUSP6 based on global proteomic analyses of A375 cells treated with 1 µM Vertex11e.

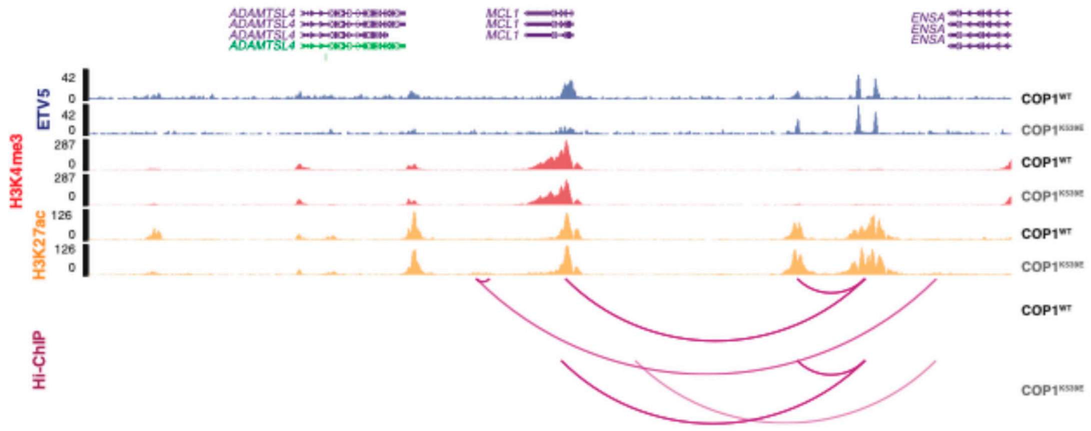

Figure S4. *COP1^{K539E}* cells have altered contacts between active enhancers and active promoter regions for *MCL1*. Traces show normalized ETV5 (blue), H3K4me3 (red), and H3K27ac (orange) ChIPseq reads around the *MCL1* gene. Hi-ChIP arcs (magenta).

Table S1. List including a collection of 542 compounds, obtained from in-house synthesis or purchased from commercial vendors, used for the chemical genomic screen in Figure 1D-E.

G-number identifier	Target/pathway
G00050939	MEK inhibitor
G02442896	ERK inhibitor
MEK inhibitor	ERK inhibitor
G02584994	ERK inhibitor
G00057455	Refametinib
G02443714	Pimasertib, MEK
G00064994	B-Raf
G00045157	PARP1, PARP2
G00033054	MEK1, MEK2
G02842573	Taxol derivative
G02665104	ERK inhibitor
G02859606	PKD1
G02848535	ERK inhibitor
G02643486	BET inhibitor
G02442404	Mannosidase
G02961433	PRMT5
G00028831	Kinase inhibitor
G02852399	DLK
G00045409	PARP1, PARP2
G02442104	MEK inhibitor
G01579365	Mitochondrial decoupler
G02663249	
G02692670	ERK inhibitor
G01534236	mTor
G02445113	TORC1 +TORC2
G02580329	TYK2 inhibitor
G02629426	HSP90 inhibitor
G02629402	Glycolysis
G02788997	CBP
G00586299	PF-3758309
G00022086	EGFR
G02663218	SJN2511
G02447286	ERK inhibitor
G02663163	S1P1
G01579689	Cytosine arabinoside; DNA synthesis
G02788290	CBP inhibitor
G02309860	HDAC inhibitor
G02845211	Cryptophycin analog
G00826142	AEM1, Nrf2 tx inhibitor

G00028831	Kinase inhibitor
G00018820	Microtubule inhibitor
G01580120	Rotenone, mitochondrial e- transport chain inhibitor
G00425652	PIM inhibitor
G02940155	Shokat G12C irreversible inhibitor
G02629396	PDK1
G02961432	
G02850310	GSK2606414
G02776352	BTK inhibitor
G02778407	RAF inhibitor
G02791211	MCP-110
G02663170	Rac
G02534405	Adenylyl Cyclase
G01954222	TEAD
G02843859	
G02790721	
G02852761	CECR2 inhibitor
G02852653	Purine analog; DNA synthesis disruptor
G02853448	HSP90 inhibitor
G02852378	
G02841815	PF-562271, FAK inhibitor
G02663200	ATM
G02692633	MI-2
G01527558	ATR, ATM
G02790238	EZH2 inhibitor
G02451652	Acetyl-CoA carboxylase inhibitor
G02655913	Abl, KDR, FGFR1, PDGFR, FLT3, LYN
G02663648	CPI-253766, KDM5A inhibitor
G02853173	LDHA
G01579648	Alkylating agent
G01579307	Proton pump
G02843881	everolimus, MTOR inhibitor
G00056977	SAR-302503
G02639727	PAK inhibitor_group 1 selective
G02629418	HSP70
G02441729	PI3K inhibitor_brain penetrant
G02846213	CARM1 inhibitor
G02095189	NPC1L1
G00044364	RAF inhibitor
G00035898	p38 MAPK
G02444247	Lilly Cdk4/6 inhibitor
G02774322	
G01527563	ALK2, ALK3, ALK6
G01579643	DNA

G02937979	HSP90
G02229893	DNA
G02845481	DLK1
G01579122	Alkylating agent
G02664757	CDK1
G01579406	DNA methyltransferase
G01580596	Anti-Androgen
G02942349	inhibitory effect on mitochondrial ATPase activity
G02845725	
G02849750	LSD1 inactive inhibitor
G00427867	Quizartinib
G02603976	CPT1A inhibitor
G00037687	p38alpha inhibitor
G02683553	Degrasyn
G02594617	Nampt inhibitor
G02629423	PLK1
G01528057	L-type calcium channel
G01527572	CDK4
G02449631	Topotecan
G02841570	PIM inhibitor
G02937977	DGAT-1
G02629412	Cdc25A, Cdc25B, Cdc25C
G00592921	Erismodegib
G03081672	CRAF
G01606145	Apoptosis inhibitor
G00026887	RAF inhibitor
G01715571	Eg5
G01579599	iPLA2 beta
G02770994	
G00025563	Aurora_A, Aurora_B
G02663169	
G01571613	SC-514, NFkB inhibitor
G00033795	PARP1, PARP2
G02663820	GLS
G02663202	p38 MAPK
G02663228	PF3845
G02777263	CDK8 inhibitor
G02636747	IGF1R inhibitor
G02001876	Cdk4 inhibitor
G02844997	ITK inhibitor
G01578588	Glucocorticoid agonist
G01578920	IMPDH
G00042409	IAP
G02582339	PIM inhibitor

G00676181	CPI-187389
G02787233	Microtubule
G02664748	20S Proteasome
G01579617	TNF-α-induced IκBα phosphorylation inhibitor
G00049868	MEK inhibitor
G02452804	PI3Kgamma/delta
G01524537	multi-Kinase
G02663162	Hec1, NEK2
G02879621	IRAK4 inhibitor
G01424126	
G02621702	Abl, Src
G02663191	Febuxostat
G02445034	G9a inhibitor
G02855555	Pak1 inhibitor
G02695746	Alprazolam
G02788290	
G02772125	ITK inhibitor
G03089751	PDE delta inhibitors
G02580172	Anti-metabolite
G02858925	
G02664746	AMPK
G02441753	Caspase
G02447892	DNA synthesis
G02841571	PIM inhibitor
G00038390	PI3K mTOR dual inhibitor
G01527613	multi-CDK
G02444500	Vitamin D Receptor
G00025694	KDR, PDGFR, Kit, Flt3
G01578558	Alcohol dehydrogenase inhibitor
G01524038	TBK1, PDK, IKK_alpha
G02445000	Romidepsin
G00588791	ROCK1, ROCK2
G02937856	CARM1 inhibitor
G02663192	Exemestane
G00425669	CHK1
G01786072	PPAR-gamma
G01579617	NFKB
G01578514	Pyrimethamine
G02069334	Cox inhibitor
G00426134	Quinine Sulfate
G01527509	DNA-PK
G00063738	G6PD
G00376771	Bcl-2 inhibitor
G02663247	AT II R

G02447946	HDAC1/2 inhibitor
G02849513	EZH2 inhibitor
G01884888	Tamsulosin
G00018828	DNA synthesis
G00031044	multi-CDK
G02192952	Topoisomerase II
G02852281	Alkylating agent
G01719102	protein trafficing inhibitor
G02748529	DNA
G02663174	Flt3, PDGFR, Kit
G0269401	STAT3 inhibitor
G01594217	p53
G01579888	PDE
G02664744	sc26196
G02512827	Muscarinic Receptor
G02663241	
G02922829	
G02663198	ACE
G02849751	LSD1 inhibitor
G02663219	PARP-2
G00056706	JNJ-38877605
G02629415	FAK
G00028527	Erbb2/EGFR inhibitor
G02663193	HDAC
G00374349	GDC-0349, MTOR
G02931611	Pak1 inhibitor
G03163506	CLK4 inhibitor ML167, Cdc2-like kinase4 (Clk4)
G02404036	NAPH oxidase
G00428179	p38 MAPK
G02663215	ACAT
G02663326	PERK inhibitor
G03089752	PDE delta inhibitors
G02774432	T790M-EGFR inhibitior
G02663226	p70S6K
G01975452	DNA synthesis
G01578839	GR
G01580023	Unknown
G02663167	bisphosphonate
G02953350	Bafetinib, CNS-9
G02695737	MDM2 inhibitor
G01427151	CLK inhibitor TG003
G00053234	Src and Bcr-Abl inhibitor
G01578535	PDE
G02695731	DNA

G00063874	JAK1, JAK2
G02778169	MI-2-2
G03090660	EGFR inhibitor (EAI045)
G02094107	SSRI
G02774446	
G02937972	17,20 Lyase
G01578599	Estrogen Receptor antagonist
G02792917	NIK inhibitor
G00039804	ALK, ROS1, MET inhibitor
G02601585	CK2
G00024977	KDR, EGFR, Ret
G02664752	26S Proteasome
G02695736	PKC Inhibitor
G00025347	26S Proteasome
G02494239	Transketolase
G02774619	ITK inhibitor
G00045533	PHA-767491
G00032264	Bcl-2 Bcl-xL inhibitor
G02663734	
G02782682	PAK inhibitor_group 1 selective
G00021479	Inhibitor of sarco/ER Ca++ ATPase (ER stress)
G02663216	RAR_alpha
G01718697	PDE
G00063418	Abl, Kit, PDGFR
G01579942	HMG-CoA reductase
G02845598	
G02953213	BMS-777607
G01764362	HMG-CoA reductase
G02599728	Axl inhibitor
G03061952	KRAS G12C
G00064905	TLR7
G03055518	ubiquitin (Ub) E1 inhibitor
G01578747	Norepinephrine reuptake
G02777186	KDM6B inhibitor
G02663214	Precose
G02861550	
G00424761	
G01525326	PI3K inhibitor_alpha-selective
G02663217	FFAR1
G01578035	GAGAa
G02670458	Proteasome inhibitor
G00461180	Eg5
G02634875	Nampt inhibitor
G01579767	CDK1, CDK2, CDK9

G02852652	Inhibitor of actin polymerisation
G02854021	L3MBTL3 inhibitor
G02859604	PKD1
G02663233	multi-Kinase
G02656306	4m8c, IRE1 RNase inhibitor
G02663221	ALK2
G02629427	topoisomerase inhibitor
G01527615	ATM
G02841815	PF-562271
G01578633	Cerebulin
G01579152	Guanylyl Cyclase, PLC
G00038960	IAP antagonist
G02663179	CDK5, CDK9
G02580499	Cysteine Protease
G02663222	Golgi
G01987812	CDK inhibitor
G01694878	Cholinesterase
G02663166	GGTase
G02663204	HSP90 inhibitor
G00018824	Topoisomerase I inhibitor
G02663157	P2Y12
G02541797	JAK2
G02777186	KDM6B
G01527511	DAG kinase
G01886396	TrioN RhoGEF
G02069152	H1 Receptor
G02601856	IRAK4 inhibitor
G02447610	HDAC 1/2 inhibitor
G02663239	PDE5
G01579311	DNA
G02663183	Necrosis inhibitor
G02854097	KDM5 inhibitor
G00018776	microtubule inhibitor
G02448614	BTK inhibitor
G02852889	EGFR inhibitor
G01578893	HMG-CoA reductase
G00025897	SMO
G00424795	nucleotide synthesis inhibitor
G02787235	Aurora Kinase inhibitor
G02663185	Na+/H+ exchanger
G00032970	multi-Kinase
G01580041	Guanylyl Cyclase
G00024365	Kit, PDGFR, VEGFR
G02594618	Nampt inhibitor

G00022931	EGFR
G02851179	
G00038448	Aurora_B
G01671767	
G02264070	
G02663647	CPI-253455, KDM5A inhibitor
G01527537	EGFR T790M inhibitor
G01578931	GR
G00050129	ALK
G02542112	MAP4K4 inhibitor
G02663223	LY311727
G01579761	ODC
G02778631	Tankyrase inhibitor
G02671049	BTK inhibitor
G01441223	Antioxidant
G01580257	HMG-CoA reductase
G01580068	
G01578781	DNA polymerase
G01579981	bNOS, eNOS
G01917140	COX-2
G02694848	GNF5837
G00449734	AKT inhibitor
G02664745	PDE5
G02542431	Tankerase inhibitor
G00374349	mTOR inhibitor
G02444526	inhibit translation initiation
G02695739	HSP90
G02663230	
G02663231	Caspase
G02441753	Belnacasan
G02579877	HDAC3 inhibitor
G01580022	oxamic acid
G01578724	Quinacrine
G02663242	DNA synthesis
G02602861	PAK inhibitor_group 2 selective
G03163508	Selinexor, KPT-330
G00696521	bisphosphonate
G02663189	BIBR1532
G00018818	Type 2 topoisomerase inhibitor
G01578864	Progesterone Receptor
G01665131	CETP
G00037546	Aurora_A
G01578865	90S Ribosome
G00913749	PPAR-gamma

G01580074	Androgen Receptor
G02937985	Eg5
G02051419	Sitagliptin Phosphate Monohydrate A
G02663213	DNA synthesis
G02937855	PRMT3 inhibitor
G02120612	DAG lipase
G02790984	BAZ2B inhibitor
G01578553	BAR
G02841166	SETD7 inhibitor
G02601845	Afatinib
G01902758	HDAC
G02858815	Wnt-C59, porcupine inhibitor
G00505032	PI3K
G02346867	
G01578915	chloroquine diphosphate
G02786240	LSD1 inhibitor
G02948255	ATF6
G02629432	Rsk1, Rsk2, Rsk3
G01578685	PPAR-alpha
G02580500	Calpain
G03061086	CB-5083, UPS and ERAD inhibitor
G02663171	Src, FGFR1
G02849852	Narciclasine, Rho/ROCK/LIM kinase/cofilin pathway
G02447145	MPS1 inhibitor
G02937973	Nedd8
G00018821	Microtubule
G01581023	LRRK2 inhibitor
G02229775	MMP
G02629597	Lipase
G02663234	bisphosphonate
G02663212	SIRT1, SIRT2
G03046709	dual NEDD8/Ubiquitin (Ub) E1 inhibitor
G02854998	
G01579860	decreases deoxyribonucleotides
G02663209	FAS
G00064665	MCP-1
G00571684	RIPK1
G02034018	Varespladib
G01579800	iNOS, eNOS, nNOS
G00043344	p53/MDM2
G02852280	Anti-microtubule agent; vinca alkaloids
G00038989	PI3Kdelta inhibitor
G01579270	Letrazole. Aromatase inhibitor
G01580026	Guanylyl Cyclase

G02663176	DNA synthesis
G01641066	CPT1b inhibitor
G00588286	PI3Kdelta
G03062332	USP7
G02664495	MAP4K4 inhibitor
G02854878	
G00027749	Akt inhibitor 1/2
G00537693	SphK
G02950761	GDC-0927, SERD
G01580114	boniva, bisphosphonate
G02444504	Androgen Receptor
G02663206	WEE-1
G02771674	LDHA/B inhibitor
G02629408	Actin
G02599853	BTK inhibitor
G02696344	EGFR-T790M selective inhibitor
G02664747	monocarboxylate transporters
G01527504	multi-Kinase
G02663188	Na+-Glucose Transport
G02695744	PLK1
G02937976	Cathepsin K
G01578780	PPAR
G02447031	Nampt inhibitor
G02663208	Antioxidant
G03163509	PF-06747775, EGFR T790M inhibitor
G01578950	COX-1, COX-2
G01580989	JAK1 inhibitor
G00458326	CTP Inhibitor
G02439349	MET inhibitor
G02663203	DNA methyltransferase
G02629422	GlcNAc phosphotransferase
G01578616	K+ channel
G01579259	SSRI
G00428024	glycolysis inhibitor, Hexokinase
G02695734	FABP
G02663181	NAMPRT
G02953217	KI20227
G00049729	AZD-7762
G01527588	multi-Kinase
G02671036	Myrexis
G02663158	Survivin
G02777056	ITK inhibitor
G00035608	Akt inhibitor
G02664754	HMG-CoA reductase

G00814558	CerK
G01580128	histone methyltransferase inhibitor
G02051419	DPP-4
G02440955	Syk inhibitor
G01579880	GluR
G00018822	Microtubule stabilizer
G01522403	SCD1 inhibitor
G00035837	PLK1
G01578613	Ornidazole
G03072255	DUSP6
G02324281	ACE
G02445420	G9a inhibitor
G01579143	ER
G00063622	COX-1, COX-2
G02663220	5-HT2 Receptor
G02663168	p38 MAPK
G02241174	MCT1 lactate/H+ symporter
G00043484	JAK inhibitor_pan
G02663248	AT II R
G02845775	PIM inhibitor
G01579246	GR
G02142909	Aromatase
G03020378	Nelfinavir Mesylate, ER Stress, UPR
G02664749	Mre11
G00590559	IRE1 inhibitor
G00018860	Microtubule
G02663229	MMP-3
G01579792	PNMT
G02695748	Topoisomerase II
G01773604	PPAR-gamma
G00052423	Syk
G00008764	COX-1, COX-2
G02443692	ER
G00041297	Cerebulin
G00037683	Pyridone-6
G01427804	SIRT1
G01578886	SIRT1
G00587034	TPCA-1
G02937975	Rac-GEF
G02664876	Iron chelation
G00018826	Topoisomerase II
G02624467	Estrogen Receptor antagonist
G00056092	Bcl-2
G02557755	Bcl-xL inhibitor

G02629866	CDK inhibitor
G02663161	COX-1
G00586305	Abl, Kit, PDGFR
G02788034	FGFR
G00038794	FGFR, KDR
G01578460	PPAR-gamma
G02395839	Muscarinic Receptor
G02664742	Topoisomerase II
G00028210	Aurora_B
G01579954	FLAP
G02663164	P-gp
G03163507	KPT-185
G00033829	PI3K inhibitor
G02034018	sPLA2
G00947044	PDE5
G00418329	Ruxolitinib
G02449967	
G01909491	HDAC
G00023251	Mycotoxin. Protein synthesis inhibitor
G02696164	Unknown
G02663177	COX-2
G01707216	Flt3, PDGFR, Kit
G02448347	Tankerase inhibitor
G01578489	Inhibitor of glucose production
G02859284	NIK inhibitor
G03020607	EGFR,HER2
G02663195	
G00018838	DNA
G00032555	dibenzazepine, g-secretase inhibitor
G01579405	HMG-CoA reductase
G02663227	L006235
G03033334	Saxagliptin
G02925614	Alectinib, ALK inhibitor
G02001876	Palbociclib, PD-0332991, CDK4/CDK6 inhibitor
G03163505	GSK923295
G02629413	Cytidine deaminase
G02950274	GDC-0810, SERD
G02786239	KDM6B inactive inhibitor
G02663225	TrkA, TrkB
G02629410	multi-Kinase
G02447171	SCD1 inhibitor
G02852889	Osimertinib, AZD9291, EGFR T790M inhibitor
G02663197	TGBR1
G02937980	Ionophore signaling

G01567836	Aurora_A, Aurora_B
G02663173	Topoisomerase II
G02663201	GSK3_beta
G00031603	ON-01910
G02663224	DNA-PK
G01579361	ACE
G00696773	p38 MAPK inhibitor
G02229089	DNA synthesis
G00587330	CHK1 inhibitor
G00022937	EGFR, ErbB2, ErbB4
G01579636	Dexamethasone
G00047010	PF-00477736
G02211155	DNA synthesis
G02663182	ACC
G00025665	KDR, PDGFR
G01527528	MET
G01579042	Iron chelation
G02601712	CHK1 inhibitor
G02539870	IC-83
G01579932	Hif1alpha
